# A Review of Methods for Research Synthesis

**DOI:** 10.1002/sim.70314

**Published:** 2025-12-04

**Authors:** Pär Villner, Matteo Bottai

**Affiliations:** ^1^ Division of Biostatistics Karolinska Institutet Stockholm Sweden

**Keywords:** data heterogeneity, effect‐size surface, meta‐analysis, research synthesis

## Abstract

Meta‐analysis consists of a wide range of methods for summarizing existing research, often by aggregating summary statistics. The dominant methods are the fixed effect and the random effects models, which assume that all studies included in a meta‐analysis are similar. In many scenarios, the available studies differ in important ways, for example, in terms of research design and sample population. To handle this heterogeneity, more advanced methods are required. In this article, we review some of these methods that have been proposed in the past decades: hierarchical models, bias adjustment and quality weighting methods, Bayesian methods, and decision‐centered meta‐analysis. We aim to describe the theoretical rationale behind the methods and to give examples of applications. Each method has advantages and limitations, and we consider ways of combining methods.

## 1 Introduction

Meta‐analysis consists of a wide range of methods that aim to summarize existing research, usually by averaging the estimates of a population quantity that are reported in different studies. The interpretation of the average estimate depends on the similarity of the studies included in the meta‐analysis. When all studies are similar in terms of study population, research design, outcome measure, and intervention measure, differences between the parameter estimates can be attributed to sampling error. In this case, one may assume that all studies estimate the same parameter, and the average of the reported estimates provides an unbiased estimate with higher precision than that of any individual study alone. In mathematical notation, the jth study reports the estimate

$$\begin{array}{l} {\hat{\theta }}_{j}∼N(\theta ,\sigma_{j}^{2}),\;j=1,\ldots ,m \end{array}$$

where θ is the true parameter, $\sigma_{j}^{2}$ is the sample variance of the jth study, and m is the number of studies. An estimate of θ is a weighted average of the estimates reported in all studies, where the weight of the jth study is $1/{\hat{\sigma }}_{j}^{2}$. This is referred to as the fixed effect model, and it is the type of analysis that is envisioned when meta‐analysis is described as the strongest type of evidence, superior to randomized clinical trials (RCTs) and observational studies [1].

In most settings, studies differ in terms of the study populations, study design, outcome measures, and interventions. In such situations, it is still possible to calculate an average of the reported parameter estimates. To account for differences between studies, a random effects model is commonly used. The random effects model assumes that all of the m collected studies are potentially estimating a unique parameter. In mathematical notation, the jth study reports the estimate

$$\begin{array}{l} {\hat{\theta }}_{j}∼N(\theta_{j},\sigma_{j}^{2}) \end{array}$$

where $\theta_{j}∼N(\theta ,\eta^{2})$. The parameter θ can be interpreted as an overall mean valid for a population of studies, and $\eta^{2}$ describes the variation between the true parameter values. From a frequentist perspective, the studies included in the meta‐analysis are a sample from this population. From a Bayesian perspective, the distribution of parameter estimates refers to the subjective uncertainty of the researcher. The true study effects in the research studies are “exchangeable,” meaning that the direction in which the true $\theta_{j}$ differs from θ is unpredictable based on the study characteristics [2]. With the random effects model, an estimate of θ can be given by a weighted average of the parameter estimates reported in the studies. A common weight of the jth study is $1/({\hat{\sigma }}_{j}^{2}+{\hat{\eta }}^{2})$ [3]. Note that if $\eta^{2}=0$, there is no between‐study variance and the above is equivalent to a fixed effect model.

For a fixed effect meta‐analysis to be applicable, the analyst must set strict standards that all included studies must satisfy. All studies that do not meet the criteria must be excluded. The random effects model enables researchers to include a wider variety of studies in their analysis. It requires, however, that studies are sufficiently similar so that exchangeability can be assumed, or that the true parameters of all studies are drawn from a common distribution. This means that even with a random effects approach, many studies investigating the topic of interest must be excluded. For instance, it is common to include only studies using a particular study design, even though studies of different designs are available. According to Davey et al. [4], 75% of all the random effects meta‐analyses in the Cochrane database included fewer than five studies. Therefore, more flexible methods to summarize existing research have been proposed. In this review article, we consider the most relevant and popular among them.

### 1.1 Search Strategies

Finding relevant articles is not a trivial task, as there is no distinct name given to the methods of interest to the present review. Previous review articles served as starting points to identify relevant categories of methods and influential methodological work. All articles citing the review articles and the influential methodological work were surveyed, as well as all the articles cited in the review articles. Searches were also made using the keywords “meta‐analysis,” “hierarchical models,” “research synthesis,” “cross design synthesis,” and “confidence profile method.” The search engines used for all searches were Web of Sciences and PubMed. In many cases, the notation used in the original article was modified to harmonize with their descriptions in the present review.

### 1.2 Decomposing Differences Across Studies

It is easy to understand the result of a fixed effect analysis performed on studies that are similar. When studies differ from each other in important ways and the random effects model is used, the result of a meta‐analysis is harder to interpret. If, as is usually the case, the studies differ in terms of study design, interventions, exposures, and outcome measures, the estimate $\hat{\theta }$ no longer estimates a particular intervention effect in a particular setting for a particular population. Instead, it is a measure of the central tendency of existing research [5]. The population for which the estimate is valid is not a population of individuals, but a population of studies, which differ along many dimensions. To the extent that the goal is to “arrive at decisions affecting clinical practice [1],” it is crucial to identify what population and circumstances the result of a statistical analysis is valid for. This requires understanding all the differences between studies.

Rubin [6] coined the phrase effect‐size surface. Assume that an estimand θ depends on the vectors X and Z, where X is a vector of variables of scientific interest (e.g., age of participants and details of the study design) and Z is a vector of variables related to compromises of the researchers (e.g., sample size and control for biases). The estimated expected value of θ that is reported in a study is a function of X and Z:

$$\begin{array}{l} E(\theta |X,Z)=f(X,Z). \end{array}$$

f(X,Z) is a multidimensional space which describes how θ varies with X and Z. Different studies are estimating different points in this space. A way of summarizing existing research is to perform an analysis with studies having different values of X and Z, in order to explore the effect‐size surface. This result could then be used to estimate the effect size for the X and Z of scientific interest. Of particular interest is the situation when all studies are performed without compromises, meaning that they have infinite sample size, no measurement errors, and so forth. Rubin denotes this ideal estimate

$$\begin{array}{l} E(\theta |X,Z=z_{0})=f_{0}(X). \end{array}$$

Since the late 1980s and early 1990s, statisticians have developed methods to integrate a wide range of research to achieve an estimate of the relevant f(X,Z). All included studies may deviate from the X and Z of interest, but by combining the studies in an appropriate way, an estimate of f(X,Z) may be achieved. Turner et al. [7] give a description of the general procedure in four steps:

1. Define the target question.
2. Write a mini‐protocol for an idealized version of each study.
3. Identify internal biases (what Rubin refers to as Z) by comparing the original study against the idealized study.
4. Identify external biases (what Rubin refers to as X) by comparing the idealized study against the target question.

Whereas X and Z in Rubin's model are multidimensional, Turner et al. consider a simpler scheme, in which a study reports an estimate of

$$\begin{array}{l} \theta +\phi_{I}+\phi_{E} \end{array}$$

where $\phi_{I}$ is an internal bias term and $\phi_{E}$ is a external bias term. Both $\phi_{I}$ and $\phi_{E}$ are assumed to be functions of many types of internal and external biases. Readers familiar with the meta‐analysis literature may know of such differences between studies simply as “heterogeneity” [8] or “biases” [9, 10].

Internal biases are ways in which the actual study differs from the idealized study, that is, ways in which the study does not fulfill its stated goals. External biases are ways in which the stated goals of a particular study differ from the target question, that is, ways in which the study explicitly attempts to answer a question which is different from the question of scientific interest. In Rubin's language, deviations from the vector of scientific interest $x_{0}$ constitute external bias and deviations from $z_{0}$ constitute internal bias.

Often, all available studies suffer from biases of various kinds. Some biases are strongly associated with particular study designs. For example, RCTs are generally considered to avoid confounding bias. In addition, RCTs are generally performed under strict protocols so that detailed information about exposure is known. However, RCTs often restrict the study population so that it does not fully overlap with the target population, and study exposure often does not align with real‐world exposure. In other words, RCTs generally have a low internal bias but suffer a high external bias [11]. Observational studies are often based on samples from a broader population, and are based on realistic applications of the treatments, but have a higher risk of suffering from biases due to confounding, measurement errors, recall bias, etc. This means that observational studies can avoid some of the external biases that RCTs suffer from, while they suffer from internal biases that RCTs have a greater potential of avoiding.

Therefore, it is not surprising that authors attempting to take biases into account often focus their attention on the distinction between randomized and observational studies. Several of the methods mentioned throughout this article are presented mainly as ways of combining randomized and observational studies. However, there are many other types of bias that both randomized and observational studies can suffer from to various degrees.

The ambition of accounting for internal and external biases, and of combining data from diverse sources, is not unique to meta‐analysis. Governmental agencies are attempting to combine results from clinical trials with information from registries. In the US, the Food and Drug Administration has launched a program to combine clinical results with “real‐world evidence [12].” In epidemiological research, biases are commonly taken into account via quantitative bias analysis [13, 14]. While such methods are useful, they are not part of the present study.

### 1.3 Auxiliary Information

Outside of the set of studies explicitly estimating a parameter of interest, there is often auxiliary data that contains information relevant to the research question. For instance, a case control study reporting the association between a pesticide and a disease can report the difference with an odds ratio, but this does not reveal the absolute magnitude of the difference unless the proportion of the population who suffer from the disease is known. An estimate of this proportion may be derived from mortality or disease registries or other public databases.

Sometimes, the information given by auxiliary data, albeit relevant, is inadequate to use with standard statistical methods in order to estimate a particular parameter. For example, when judging the effect of the pesticide in real‐world applications, one may consider information regarding how the pesticide is applied in farming and industry by considering qualitative studies and information regarding typical application practices. One may also want to consider biological theories regarding how the pesticide could have a damaging effect on humans. The methods discussed in this article can use auxiliary information to varying degrees.

### 1.4 Meta‐Regression, Network Meta‐Analysis, and Chains of Evidence

Meta‐regression and network meta‐analysis are two popular extensions of the fixed effect and random effects models that enable the researcher to take a broader range of studies into account. We mention them briefly in the following two sections without giving an exhaustive summary, mainly because these methods are established and several excellent review articles are already available on both meta‐regression [15, 16, 17] and network meta‐analysis [18].

#### 1.4.1 Meta‐Regression

Meta‐regression assumes that the true parameter estimate of a study can be described by a linear function of the characteristics of the study, for example, the dose of a treatment, the mean age of the study participants, publication year, or the sampling procedure. A meta‐regression model can be used to explore how the parameter of interest varies with different values of study characteristics.

While there exists fixed effect meta‐regression, the most common approach is a random effects meta‐regression, where much of the observed heterogeneity is explained by study characteristics that are not included in the model. A simple formulation of the meta‐regression model is

$$\begin{array}{l} {\hat{\theta }}_{j}∼N(\theta_{j},\sigma_{i}^{2}) \end{array}$$

$$\begin{array}{l} \theta_{j}∼N(\beta x_{j},\eta^{2}), \end{array}$$

which can be reformulated as

$$\begin{array}{l} {\hat{\theta }}_{j}∼N(\beta x_{j},\sigma_{j}^{2}+\eta^{2}). \end{array}$$

$x_{j}$ is a vector of characteristics for the jth study and $\sigma_{j}^{2}$ represents the sampling variability of the jth study. $\eta^{2}$ is the equivalent of between‐studies heterogeneity, that is, it describes how the true parameter of a given study differs from $\beta x_{j}$ due to uncontrolled factors. The inverse of $\sigma_{j}^{2}+\eta^{2}$ serves as the weight of the studies in the regression analysis, meaning that studies with more precise estimates contribute more to the analysis.

Any characteristic of the studies may be used as a covariate in a meta‐regression analysis, including demographic characteristics of the sample population, characteristics of the intervention investigated, such as the dose of a treatment, and characteristics of the study design. Importantly, the population for which the estimate is valid is a population of research studies, not a population of patients. For instance, the mean age of study participants may be a covariate in a meta‐regression analysis, but the parameter related to mean age does not describe the association between age and the outcome for actual patients. Rather, the parameter describes the association between participants' age and the outcome estimate. It is possible that increased mean age is associated with a higher parameter estimate across studies, whereas each study reports a negative association between age and the outcome of interest [18].

#### 1.4.2 Network Meta‐Analysis and Chains of Evidence

Network meta‐analysis, also called mixed treatment comparison, can be used when estimating the difference between treatments by including studies that do not directly compare the treatments [19]. In the simplest case, when interventions A and B are compared against C in different studies, network meta‐analysis is performed by conducting separate meta‐analyses of A against C and B against C, and an estimate of the relative effect of A against B is the difference between the two averages [20, 21]:

$$\begin{array}{l} {\hat{\mu }}_{AB}={\hat{\mu }}_{AC}-{\hat{\mu }}_{BC}. \end{array}$$

${\hat{\mu }}_{AC}$ and ${\hat{\mu }}_{BC}$ are said to be transitive when ${\hat{\mu }}_{AC}-{\hat{\mu }}_{BC}$ is an unbiased estimate of ${\hat{\mu }}_{AB}$ [22]. If the studies are similar in all respects except for the treatments given or if they are at least exchangeable, transitivity holds [18, 23]. In the language of this article, whenever studies do not differ in terms of the internal and external biases, transitivity holds, and the evidence provided by a network meta‐analysis is as good as direct evidence provided by a standard meta‐analysis. While this is rarely the case in real applications, the problem is conceptually the same as when performing a standard meta‐analysis [24].

Related to network meta‐analysis is the so‐called chains of evidence analysis. It is common that a prospective study does not measure the outcome of actual interest, but a surrogate outcome that is thought to be associated with the outcome, that is, an indicator that the outcome will happen. Other studies may report on the association between the surrogate outcome and the outcome of interest. The two types of studies can be connected in a chain of evidence analysis to establish the association between an exposure and the outcome of interest [25, 26, 27, 28].

### 1.5 Previous Reviews

The difficulty of handling biases in meta‐analysis has been known for a long time, and attempts to solve the issue have been suggested since the late 1980s. Higgins and Whitehead [29] provide an early review of such methods. They discuss the standard random effects meta‐analysis, the use of Bayesian prior distributions, and network meta‐analysis. Ades and Sutton [2] provided an updated overview focused on the confidence profile method, cross‐design synthesis, hierarchical models, and network meta‐analysis. Nine years later, Verde and Ohmann [30] and Kaizar [31] published reviews. Both focus on applications where the goal is to combine randomized and observational studies, although it is mentioned that the methods can be used for wider purposes. The methods covered are largely the same as in Ades and Sutton [2]. Nikolaidis et al. [9] provided a review that categorizes methods based on how they relate different studies to each other (functional, exchangeable, multivariate, and prior‐based relationships). The authors also comment on the methods used to take different types of biases into account.

This is an updated review, dividing the methods into four broad categories and further subcategories. In contrast to Nikolaidis et al., the focus is on describing how the methods have been applied. While most of the methods have been mentioned in the earlier review articles, this review gives an updated perspective and also describes two categories of methods that have not been part of the earlier reviews.

## 2 Methods

### 2.1 Hierarchical Models

Hierarchical models are extensions of the random effects model, for situations when the included studies can be divided into groups based on characteristics, such that the exchangeability assumption of the parameter estimate can be assumed both within each group and between the groups. In terms of internal and external biases, each study type can be assumed to have a mean parameter

$$\begin{array}{l} \theta_{t}=\theta +\phi_{It}+\phi_{Et}, \end{array}$$

where $\phi_{It}$ and $\phi_{Et}$ describe the deviations from θ due to the average external and internal biases associated with the study type. Unlike meta‐regression, which attempts to disentangle the study features that lead to particular directions of the biases, the hierarchical model treats the biases as unexplained features of the study type. The purpose of the hierarchical model is to allow information sharing across different study types while treating the true parameters of the different study types as distinct. This is achieved by adding between‐study‐type variation to the between‐study variation. The between‐study‐type variation accounts for the fact that groups of studies have unique mean parameters, due to their characteristics. However, the study types are considered exchangeable. This means that the differences between study types are considered to be drawn at random from a probability distribution. An alternative phrasing is that while acknowledging that study types are different, there is no a priori reason to assume any particular difference between the study types. For instance, one may think that the average intervention effect of a treatment is different for randomized and observational studies, but there may be no particular reason to believe that the average is higher for one of the study designs. While there are unique mean values for each study type, they are linked to each other through the probability distribution they are assumed to be taken from. Therefore, an average parameter estimate across studies can be obtained, along with estimates of the between‐study and between‐study‐type variance (Table 1).

**TABLE 1:** This table lists articles mentioned in relation to each of the methods.

Method	References
Hierarchical models	Prevost et al. [32], Efthimiou et al. [20], Roh et al. [33], Schmitz et al. [34], McCarron et al. [35], Au and Cheung [36], Peters et al. [37], De Angelis et al. [38], Gamalo‐Siebers et al. [39], Schmitz et al. [40], Wu et al. [41]
Bias adjustment and quality weighting	Spiegelhalter and Best [42]; Berare and Bravo [43], Seo et al. [44], Turner et al. [7], Doi et al. [45, 46], Schnell‐Inderst et al. [47], Thompson et al. [48], Hamza et al. [49], Wilks et al. [50, 51], Dias et al. [52, 53], Salanti et al. [54], Fu and Lin [55], Wolpert and Mengersen [56], O'Rourke et al. [57, 58], Greenland [59, 60], Conde et al. [61], Trinquart et al. [62], Welton et al. [63], Rhodes et al. [64], Verde [65], McCarron et al. [35], Efthimiou et al. [20], Wheaton et al. [66], Yao et al. [67], Raices Cruz et al. [68], Goto et al. [69], McCandless et al. [70], Mathur and VanderWeele [71], Walker et al. [72], Wheaton et al. [73].
Bayesian priors	Morfaw et al. [74], Salpeter et al. [75], Knight et al. [76], Turner et al. [77], Ren et al. [78], Gamalo‐Siebers et al. [39], Schmitz et al. [40], Efthimiou et al. [20], Proctor et al. [79], Schmidli et al. [80], Mutsvari et al. [81], Ibrahim and Chen [82, 83], Rietbergen [84], Hatswell [85], Jenkins et al. [86], Hong et al. [87], Hobbs et al. [88], Prevost et al. [32], Jones et al. [89], Bartell et al. [90]
Adjustment for model‐misspecification bias	Kundu et al. [91], Zhang et al. [92], Ray et al. [93], He et al. [94], Huang et al. [95], Chatterjee et al. [96], Thom et al. [97]
Adjustment for sample selection bias	GAO [98], Kaizar [99], Hidaka et al. [100], Vaitsiakhovich et al. [101], Wilkes et al. [102], Tsuda et al. [13], Lauen et al. [103], Degtiar and Rose [10], Dahabreh et al. [104], Zuo et al. [105], Barker et al. [106], Vo et al. [107], Phillippo et al. [108], Yang et al. [109], Han et al. [110], Li et al. [111], Hu et al. [74]
Decision‐centered meta‐analysis	Manski [112]

An example is Prevost et al. [32], where RCTs and observational studies are considered two separate study types with different true parameter estimates related according to the model

$$\begin{array}{l} {\hat{\theta }}_{jk}∼N(\theta_{jk},\sigma_{jk}^{2}) \end{array}$$

$$\begin{array}{l} \theta_{jk}∼N(\theta_{k},\eta_{k}^{2}) \end{array}$$

$$\begin{array}{l} \theta_{k}∼N(\theta ,v^{2}), \end{array}$$

where j refers to study and k refers to study type, that is, $\theta_{jk}$ is the true parameter of the jth study of study type k.

Many characteristics can be used to divide studies into groups. A common characteristic is study design, where one group consists of randomized studies and another group consists of observational studies, see Prevost et al. [32], Efthimiou et al. [20], Roh et al. [33], Schmitz et al. [34], McCarron et al. [35], and Au and Cheung [36]. Another characteristic is the study population. Peters et al. [37] build a hierarchical model where one group of studies is studies with human participants, and a second group consists of animal studies. Gamalo‐Siebers et al. [39] discuss an application where one group of studies is based on adult participants and another group of studies is based on pediatric participants. De Angelis et al. [38] estimate the prevalence of HIV in several subpopulations. Raymond et al. [113] and Conti et al. [114] present similar applications. Schmitz et al. [34] perform a meta‐analysis summarizing health economic evaluations, and divide the studies into clusters based on what instrument is used to measure the economic costs and benefits.

Hierarchical models yield estimates of both the study type parameters $\theta_{k}$ and the overall mean θ. One question is whether θ is of any practical interest, or if it is rather a hyperparameter that is not relevant for an actual population. The latter seems true for some applications. For instance, if one study group has human participants and another group animal participants, the overall mean seems to be relevant for neither humans nor animals. In other settings, a possible answer is that different types of studies tend to suffer from biases of different kinds that may outweigh each other when we average their means. For instance, randomized studies are assumed to suffer less from unmeasured confounding, but at the same time, they tend to include only a subset of the target population. Observational studies, on the other hand, are assumed to be based on a sample more representative of the target population, while unmeasured confounding is a problem. If these different biases cancel each other out, the mean of the two biased estimates would yield an unbiased estimate of the parameter of interest. As Kaizar [31] suggests, this would be the case if one study type is an underestimate and the other study type is an overestimate, and if the magnitude of the errors corresponds to the assigned weights.

If interest is in the $\theta_{k}$ parameters, the question is why a hierarchical model should be used rather than performing separate meta‐analyses for each study group. The answer is that hierarchical models allow for “information sharing” across study types, due to the fact that they are related via a common probability distribution. This means that the estimate of $\theta_{k}$ is trustworthy under the assumption that the hierarchical model is correct, that is, assuming that the different study types are centered around θ according to a $N(\theta ,v^{2})$ distribution. If the distributional assumption is not met, the estimate of $\theta_{k}$ will be biased, as the estimates are shrunk toward the overall mean. While the normal distribution is very common in hierarchical models, it is possible to assume different distributions. For instance, Wu et al. [41] suggest a skewed normal distribution, which adds a parameter to take skewness into account.

A factor that limits the hierarchical model's ability to accommodate internal and external biases is that there are usually only a handful of study types included in a hierarchical model, meaning that the between‐study‐type variance parameter has to be based on very few $\theta_{k}$. To achieve a reasonable estimate of the between‐study‐type variance, a Bayesian approach can be used, where the prior distribution of the parameters plays an important role. All examples of hierarchical models that we have found are formulated within the Bayesian framework. The use of priors is discussed in the section of this paper on prior distributions. In situations where many types of studies are available, however, the hierarchical model can be extended with additional levels, thereby exploiting more dimensions of bias. Peters et al. [37] develop a hierarchical model where, on top of the level with animal and human populations, there is a level for the animal species included in the animal studies. In general, however, it is difficult to use deeper hierarchical structures due to the limited number of available studies. A more common solution is to use other tools discussed in this article to handle different biases. We return to this in Section 3.

### 2.2 Bias Adjustment and Quality Weighting

Assume that the jth study reports

$$\begin{array}{l} {\hat{\theta }}_{j}=\theta +\phi_{Ej}+\phi_{Ij}+\epsilon_{j}, \end{array}$$

where θ is the parameter of interest, that is, in Rubin's notation it is $E(\theta |x,z_{0})$ for the x of interest; $\phi_{Ej}$ is an external bias term; $\phi_{Ij}$ is an internal bias term and $\epsilon_{j}$ is a sampling error term. If $\phi_{Ej}$ and $\phi_{Ij}$ are known, it is natural to correct for them to obtain an unbiased estimate of θ. Usually, $\phi_{Ej}$ and $\phi_{Ij}$ are not known. We can then consider them to be random variables with a mean and variance. The parameter estimate can be corrected for the biases by subtracting our best estimates of the bias terms (e.g., the mean values). We refer to this as bias adjustment, related to quantitative bias analysis in the context outside of meta‐analysis. Lash et al. [11, 115] discuss methods sharing similarities with those described in this section.

When bias adjustment is performed, the biases remain problematic because they introduce additional uncertainty regarding the parameter estimates. Weighting is a way of handling the uncertainty. Less weight is given to studies with more bias. This makes sense to the extent that studies with more bias are also assumed to have more variance. In the case of internal bias, this is often a plausible assumption. When a study lacks scientific rigor, for instance, because the sample size is low, it runs a higher risk of under‐ or overestimating the true treatment effect in unpredictable ways. Studies with less rigor can therefore be expected to suffer from more variance.

There is a strong connection between bias adjustment and weighting. Most weighting approaches consider internal biases and assign weights based on rigor. Authors who take external biases into account mainly do so through bias adjustments. When external biases are assumed to show greater variance or when internal biases are assumed to have a direction, both weighting and bias adjustments can be performed. We choose to treat both bias adjustment and weighting in the same section.

Broadly speaking, there are three ways of assigning weights and estimating the magnitude of bias: common sense and expert opinion; external data sources; and statistical modeling. It may appear obvious that estimates based on data are superior to subjective judgments from researchers or experts. However, it is rare that relevant information regarding biases is available for all studies, which means that there will often be a limit to how much adjustment can be made with external data. Turner et al. [116] discuss the difference between quality weights based on expert opinion and empirical evidence. See also Vale et al. [117] for a discussion on the reliability of bias evaluations. Basing the bias adjustments on expert opinion means that the adjustment can be based on a broad set of data and considerations, as the opinion of a subject‐matter expert is presumably based on a wide range of data and scientific theory. The risk, of course, is that the experts are wrong in some of their assumptions, and then the bias‐adjusted summaries perpetuate these mistakes. Statistical modeling offers more flexibility in that the bias terms used are also shaped by considerations of model fit. Most of the applications discussed in this section use either expert opinion or external data to provide better evidence on plausible values for the bias term, often in the form of a prior distribution. With little available data, as is often the case, we run the risk of overfit by introducing additional parameters.

In this section, we will first discuss the three methods for assigning weights and estimating the magnitude of bias. Thereafter, two common types of bias adjustment are discussed: adjustment for model‐misspecification and sample selection bias. We do not mention publication bias, which is perhaps the most discussed form of bias in meta‐analysis. Much has been written in this area, and there are already excellent review articles, for instance Sutton et al. [118].

#### 2.2.1 Adjustments Based on Expert Opinion

Spiegelhalter and Best [42] presents a significant example of how to design quality weights reflecting internal biases. The authors assume that the true parameter of the jth study is given by

$$\begin{array}{l} \theta_{j}=\theta +\phi_{Ej}+\phi_{Ij}. \end{array}$$

Both $\phi_{Ej}$ and $\phi_{Ij}$ are considered normally distributed random variables with variances $\sigma_{E}^{2}$ and $\sigma_{Ij}^{2}$. The variances for the external biases are the same for all studies, meaning that the studies are considered exchangeable with regard to external bias. The internal biases differ between the studies, reflecting the rigor with which they have been performed. Assuming the biases are either adjusted for or that their expected value is zero, the overall average parameter estimate is calculated using the weights

$$\begin{array}{l} q_{j}=\sigma_{E}^{2}/(\sigma_{E}^{2}+\sigma_{Ij}^{2}), \end{array}$$

where $\sigma_{Ij}^{2}$ represents the variance of the internal bias for the jth study. This means that a rigorous study with no internal biases ($z_{0}$ in Rubin's language) is assigned a weight of $q_{j}=1$, whereas less rigorous studies with internal biases are given a weight of less than one. Spiegelhalter and Best assign the quality weights $q_{j}$ solely based on study design. RCTs are assumed to have no internal biases and are given a weight of 1, whereas registry‐based studies are given a weight of 0.5, and case series studies are given a weight of 0.2. Berare and Bravo [43] and Seo et al. [44] propose similar adjustments for quality.

Turner et al. [7] assume a model similar to Spiegelhalter and Best, but they assign weights to each study based on both internal and external biases. Since both the internal and external variances differ between studies, the mathematical definition of the weights in Turner et al.'s model is more complicated, and we do not reproduce it here. Weights are assigned by letting expert assessors compare each study to an idealized study with regard to a list of potential biases, both internal and external. Based on this, the assessors give an estimate of the magnitude and direction of both internal and external bias, which is then used to adjust the biases and form the weights for each of the studies. See also Doi et al. [45, 46].

The weighting scheme proposed by Turner et al. has been influential. Similar methods are used in applied studies. Schnell‐Inderst et al. [47] estimate biases in studies according to expert judgment of data on the effect of total hip replacement. Thompson et al. [48] synthesize studies on the correlation between physical activity and obesity; weights are assigned by clinical experts and statisticians. Hamza et al. [49] use quality weights assigned by experts in the context of meta‐regression. Wilks et al. [50, 51] perform bias adjustment and weighting based on expert judgment using dietary data. Dias et al. [52] propose a bias‐adjustment model within network meta‐analysis where the direction of the bias changes depending on what interventions are being compared – bias is assumed to favor the active intervention against placebo, or to favor the newer intervention, or the intervention marketed by the study sponsor. The authors apply the method to studies on interventions against caries in children. See also Dias et al. [53] and Salanti et al. [54] FuandLin [55] propose a random effects model where the true parameter values of each study are assumed to follow a skewed normal distribution. The authors then create quality weights based on study rigor and between‐study variability as well as the skewness parameter describing the shape of the distribution of the bias terms.

#### 2.2.2 Adjustments Based on External Data

There are several examples of methods for adjusting and weighting studies for bias based on external evidence. In some cases, there is evidence on how particular biases distort the estimates. Wolpert and Mengersen [56] analyze studies on the effect of passive smoking on cancer. Each study is evaluated based on how much effort the authors spent to prevent misclassification of passive smokers. To estimate the magnitude and direction of the biases, Wolpert and Mengersen use reports conducted by the Environmental Protection Agency on the magnitude of the different types of classification bias. Each study is then adjusted by subtracting the estimated bias terms from the reported estimates. O'Rourke et al. [57, 58] have written a series of meta‐analyses where inference is based on observational studies. The reported estimates are adjusted to account for the fact that, according to separate studies on the self‐reporting bias, the median observational study overestimates the treatment effect by 30%. Conde et al. [61] estimate the prevalence of ALS in Portugal by using records of prescriptions of medicines commonly used against ALS. However, to adjust for non‐compliance of drug users and for the fact that some ALS patients are non‐users of the medications, the authors use additional data sources to obtain estimates of these bias terms.

As Wolpert and Mengersen notes there is, in general, little available evidence on how a particular deficiency in studies distorts estimates. For such situations, an alternative approach is to estimate the effect of biases by analyzing a broader range of data. An example of this is Welton et al. [63] who propose to use weights and adjust biases based on meta‐epidemiological data. Studies are defined as having a low or high risk of internal bias, based on the presence or absence of particular design flaws. For high‐risk studies, the observed estimate is

$$\begin{array}{l} {\hat{\theta }}_{j}∼N(\theta +\beta_{j},\sigma_{j}^{2}) \end{array}$$

where $\beta_{j}$ is a bias parameter that is assumed to follow the same distribution for all high‐risk studies included in the meta‐analysis, with a common mean value $b_{m}$:

$$\begin{array}{l} \beta_{j}∼N(b_{m},\eta^{2}). \end{array}$$

$b_{m}$ is valid for the meta‐analysis in question, and it is in turn considered sampled from a larger population of mean biases, such that

$$\begin{array}{l} b_{m}∼N(b_{0},\phi^{2}). \end{array}$$

$b_{0}$, $\phi^{2}$, and $\eta^{2}$ are estimated based on meta‐epidemiological evidence, namely high and low risk studies. When the results of the high‐risk studies have been adjusted for, a random effects meta‐analysis is performed where the high‐risk studies are given less weight, corresponding to the additional variance $\eta^{2}$ that they suffer from.

Welton et al.'s approach assumes that the biases of studies are exchangeable. A more flexible approach is presented by Rhodes et al. [64] Meta‐epidemiological data is divided into categories based on the risk of bias, and for each category an empirical distribution function of the bias is fitted. Weights in a meta‐analysis can then be created based on the empirical distribution of the category the study in question belongs to. Rhodes et al. extend this approach with one that asks experts to estimate the bias of studies based on expert judgment, and uses the empirical distribution functions as prior distributions. Greenland [59, 60] uses a Bayesian framework where several types of internal bias are modeled individually. Auxiliary information is used to model the prior distribution of the bias parameters.

#### 2.2.3 Adjustments Based on Statistical Modeling

It is natural to try and model the impact of biases on parameter estimates. When information about the potential sources of bias in each study is detailed, meta‐regression techniques can be used [53]. The methods discussed below are adapted to situations when information about the characteristics assumed to cause the bias is vaguely described for each study, as is often the case.

Verde [65] assign a weight of $q_{j}$ to each study, representing the proportion of between‐study variation not explained by internal bias, as well as a parameter $\pi_{bias}$ representing the probability of internal bias. Using a Bayesian perspective, the observed parameter estimate of the jth study follows a mixture distribution

$$\begin{array}{l} \theta_{j}^{B}∼(1-\pi_{bias})N(\mu ,\tau^{2})+\pi_{bias}N(\mu_{biased},\tau^{2}/q_{j}), \end{array}$$

where in our notation, $\mu =\theta +\phi_{E}$ and $\mu_{biased}=\theta +\phi_{E}+\phi_{Ij}$. To make the bias parameter identifiable, the bias in all studies is assumed to belong to a distribution with a common mean magnitude. Rather than assigning values to the weights based on common sense or expert knowledge, Verde considers the weights to be random variables distributed as $q_{j}∼Beta(v,1)$ and the probability of bias is considered a random variable $\pi_{bias}∼Beta(a_{0},a_{1})$. Uninformative priors or priors based on previous studies can be used for the hyperparameters v, $a_{0}$, and $a_{1}$. The method is applied to data on the effectiveness of vaccination against invasive pneumococcal disease. A similar method is proposed by Yao et al. [67] The true parameter value of the jth study is considered to be sampled from

$$\begin{array}{l} \theta_{j}∼(1-\pi_{bias})N(\theta ,\tau^{2})+\pi_{biased}N(\theta +\theta_{Bj},\tau^{2}/\omega_{j}) \end{array}$$

where $\pi_{j}$ is the probability of bias of the jth study; $\theta_{Bj}$ is the bias term of the jth study; and $\omega_{j}\in (0,1)$ is an inflation factor parameter that downweighs biased studies.

McCarron et al. [35] extend the hierarchical random effects model by modeling biases due to differences between study arms in individual studies. The bias parameters are based on data from studies that report on the distribution of confounding variables, and imputed for the remaining studies. See also McCarron et al. [119].

In the context of network meta‐analysis, Efthimiou et al. [20] propose a random effects model for combining randomized and observational studies, which introduces a bias term and quality weighting term. The observed parameter value of the jth study is

$$\begin{array}{l} {\hat{\theta }}_{j}∼N(\theta_{j}+\beta_{j},{\hat{\sigma }}^{2}/\omega_{j}), \end{array}$$

where $\theta_{j}∼N(\theta ,\tau^{2})$ and $\beta_{j}$ is a bias term. All randomized studies are assumed to have $\beta_{j}=0$ and $\omega_{j}=1$, meaning that they are unbiased and not down‐weighed. Wheaton et al. [66] propose a model for a similar purpose: when one group of studies is based on population A, a second group of studies is based on population B, and a third group of studies is based on a mixture of the two populations. The authors use an extension of the random effects model, where the true intervention effect of study j can be described as

$$\begin{array}{l} \gamma_{j}+p_{j}\beta_{j}, \end{array}$$

where $p_{j}$ is the proportion of B members in the sample, $\gamma_{j}$ is the mean for population A, and $\beta_{j}$ describes how the mean in B differs from $\beta_{j}$. Both $\gamma_{j}$ and $\beta_{j}$ are considered sampled from populations of parameter values, following the logic of random effects models.

In the absence of external evidence, bias models rely on the assumptions of the analyst. Several authors make sensitivity analysis a vital part of their weighting and bias adjustment, to see under what assumptions results hold. Raices Cruz et al. [68] propose to use quality weights $q_{j}$, defined as in Spiegelhalter and Best, that are taken from a specified set of quality weights. The difference between lower and upper limits of the parameter estimates that are calculated using the different quality weights represents the uncertainty of the quality weight assignments and can be used to evaluate the results. Goto et al. [69] assume that each study has a single source of potential bias, namely confounding due to comorbidity, which affects the parameter being estimated, a risk ratio, depending on the prevalence in the exposed and unexposed group and its association with exposure and the outcome of interest. In Goto's study, the bias is assumed to have the same effect across all studies, and a range of plausible values for the bias parameters is used in a sensitivity‐like analysis. McCandless et al. [70] assume that all included studies suffer from an unmeasured confounder. The prevalence of the confounder in the exposed and unexposed groups, as well as the association with the outcome, differ between studies. McCandless et al. specify a prior distribution for the hyperparameters and perform a sensitivity analysis rather than specifying the bias term for each individual study. Mathur and VanderWeele [71] perform a similar sensitivity analysis, exploring under what assumptions regarding the bias term that meaningful effect sizes can be observed. Walker et al. [72] propose to combine data from adults and pediatric patients by assuming a bias term λ that describes the difference in the relative risk of a treatment between children and adults. While interest is in the pediatric cohort, the data on the adult patients can be used once the bias has been adjusted for. Estimates of λ are achieved through Bayesian modeling, where are range of informative prior distributions is tested. Wheaton et al. [73] propose a similar bias model to account for the mismatch between surrogate events and real outcomes in studies of colorectal cancer.

#### 2.2.4 Adjustment for Covariate‐Adjustment Bias

One common problem in meta‐analyses is that different studies use different statistical models when estimating the parameter of interest. For instance, one study may measure the association between smoking and cancer adjusted for age, whereas another study adjusts for sex. We can view such differences as an external bias. We are interested in

$$\begin{array}{l} E(\theta |x,z_{0}) \end{array}$$

for a particular x, where x includes information about study design and population, but also about the statistical model being used. Studies which are reporting estimates from a different model are hence deviating from x. If the jth study reports an estimate from a different model than the one of interest, it reports $\theta +\phi_{Ej}$ where $\phi_{Ej}$ is an external bias parameter. While such bias could be adjusted for with the methods mentioned above or with Bayesian priors (next section), this subsection covers ways of handling bias due to the model specification that exploit information that can be obtained from the algebraic structure of the models and how they are related to each other. The methods also rely on access to individual participant data, differentiating them from the rest of the methods in this review, which only require access to summary statistics.

Kundu et al. [91] propose a method called generalized meta‐analysis based on the generalized method of moments. Interest is in θ from a model f(Y|θ,X), where θ and X are vectors. Assuming that no study reports on the full model, but that all available studies report estimates from models nested within the larger model that jointly cover all θ parameters, θ can be estimated by solving a set of equations using the generalized method of moments. An illuminating example is when interest is in estimating $E(Y|X_{1},X_{2})=\beta_{0}+\beta 1X_{1}+\beta_{2}X_{2}$, and available studies are reporting parameters from $E(Y|X_{1})=\alpha_{0}+\alpha_{1}X_{1}$ and $E(Y|X_{2})=\eta_{0}+\eta_{1}X_{2}$. To obtain an unbiased estimate, the studies have to be based on samples from the same underlying population and be similar with regard to all other external and internal biases. Kundu's approach also requires a sample of individual participant data from the target population to estimate the distribution of the X variables. Zhang et al. [92] propose a similar method to achieve a more efficient estimate of θ based on individual participant data, by including a summary statistic estimate of a related variable β, such that E(g(Y|β,θ,X))=0. Zhang et al. show that including these estimates in the analysis improves the efficiency of the estimator of the full model.

Ray et al. [93] suggest a method that can be used when some studies report both an adjusted and unadjusted parameter estimate, while other studies report only the unadjusted parameter estimate. Assuming that the effects of the confounding variables that are adjusted for are the same across populations in all studies, the authors propose a method to impute adjusted estimates in the studies reporting unadjusted estimates. In the case of only two studies, this is done via

$$\begin{array}{l} {\hat{\theta }}_{1,adj}={\hat{\theta }}_{1,unadj}+{\hat{\theta }}_{2,adj}-{\hat{\theta }}_{2,unadj} \end{array}$$

where the numbers in the indices refer to study 1 (reporting only an unadjusted parameter estimate) and study 2 (reporting both an adjusted and an unadjusted estimate).

Thom et al. [97] develop a method to summarize survival data when it is reported in different formats. For instance, one set of studies may report the total number of several event types across patients, whereas another set of studies reports the total number of participants who experienced at least one type of event. The authors develop a shared parametric log hazard model that can account for all summary format types.

There are also a large number of suggestions for how auxiliary data can be used to improve efficiency in parameter estimation for specific parametric models. He et al. [94] propose a method to use Kaplan‐Meier estimates of survival probabilities to improve estimates for additive hazards models. Huang et al. [95] propose a similar method to use survival time data from registries to inform a Cox proportional hazards model. Chatterjee et al. [96] develop a method for estimating a semi‐parametric maximum likelihood regression model using individual participant data, but also summary statistics.

#### 2.2.5 Adjustment for Sample Selection Bias

A particular type of external bias is with regard to differences in characteristics between the target population and the study population. The target population is the population that the researchers are interested in; the study population is the population which the study sample was taken from. Differences in characteristics between the study and target population are external biases when these characteristics are associated with the outcome. The bias resulting from differences between study populations is sometimes called sample selection bias, and it is a term we will use throughout this section [120].

Sample selection bias is a problem in much of biostatistics and epidemiology, as the study population rarely coincides perfectly with the target population. In particular, RCTs often suffer from sample selection bias, since such trials can have strict criteria for who is allowed to participate. In a meta‐analysis, sample selection bias is a common problem, as studies have often taken samples from different populations. Some authors solve this by assuming that different populations are exchangeable with regard to the parameter of interest and use a random effects model to estimate an overall mean parameter, valid for all study populations [121]. When the goal is to estimate a parameter valid for a particular target population, the sample selection bias can be considered yet another type of bias, and some of the bias adjustment methods already discussed may be used [15, 32, 37]. In addition, authors have developed methods for bias adjustments particular to sample selection bias. A source of inspiration is the causal inference literature that use individual participant data from randomized studies together with information regarding the distribution of the relevant variables in the target population, in order to transport estimates to the target population. This is often done with weighting or matching techniques, such as propensity score weighting or matching, that adjust for the fact that the relevant variables have different distributions in the study sample and the target sample [122]. Alternatively, a regression model can be fitted based on a sample of data, and this model can then be used to estimate the parameter of interest to a target population of interest [10]. Such methods assume that there is access to individual participant data in at least the study population and that there is sufficient overlap between the study population and target population for weighting or matching to be possible, or for the regression model to be applicable. In a meta‐analytical context, methods for transporting or generalizing results to target populations that only require summary statistics have been suggested.

Cross‐design synthesis is a method developed by the US General Accounting Office (GAO) [98]. It is used to combine results from observational and randomized studies on different study populations. The idea is to combine pieces of evidence that complement each other by having different strengths and weaknesses. Whereas a randomized study is assumed to give an unbiased estimate of a particular subpopulation, this subpopulation is assumed to rarely coincide with the target population, for reasons mentioned above. An observational study, like for example those based on public registries, is assumed to cover the whole target population, but to suffer from internal biases, for example, because treatment assignment is affected by confounders. By combining the two types of evidence, an unbiased estimate of the parameter of interest for the relevant target population can be obtained. This can take the form of an “empty cell” problem. Assume that the target population can be divided into subpopulations A and B. Assume also that a randomized study included A only, while an observational study reports estimates based on both A and B. We are then lacking an unbiased estimate of subpopulation B. GAO suggests that this parameter is estimated by assuming that the ratio of the parameter estimates would be the same in the randomized study as in the observational study in both subpopulations. Hence, the natural estimate of the missing parameter is

$$\begin{array}{l} {\hat{\mu }}_{Bo}({\hat{\mu }}_{Ao}/{\hat{\mu }}_{Ar}), \end{array}$$

where ${\hat{\mu }}_{Ao}$ and ${\hat{\mu }}_{Bo}$ are the estimates of the parameter for subpopulations A and B based on the observational data, and ${\hat{\mu }}_{Ar}$ is the estimate based on the randomized data.

The report on cross‐design synthesis by GAO is sparse on mathematical details, and it does not provide a worked‐out application on real data. Kaizar [99] attempted to remedy this. Rather than simply assuming that the treatment effect is proportional in both the randomized and observational settings, Kaizar sets up a system of equations for the average effect of treatment in the full population. Solving this system of equations gives estimates of a subpopulation that was not included in the RCT. Recent years have seen several applications of cross‐design synthesis, as it is presented by Kaizar. Hidaka et al. [100] use cross‐design synthesis to estimate the effectiveness of a treatment against induced gastric bleeding in antithrombotic drug users, a subpopulation that has not been included in randomized studies but that has participated in observational studies. Vaitsiakhovich et al. [101] use a cross‐design synthesis to estimate the efficiency of a long‐term contraceptive on females younger than 20, a cohort that was excluded in an RCT but that was included in an observational study. For other applications of cross‐design synthesis in a medical context, see Wilkes et al. [102] For an application in the social sciences, see Lauen et al. [103], who investigate the effect of early college high schools on student achievements in the USA, by combining data from a randomized study with registry data.

Cross‐design synthesis as presented by the GAO and Kaizar assumes that the differences between the subpopulations used in the randomized and the observational studies are clearly defined. This is the case when a subpopulation is excluded from an RCT through inclusion criteria, but it may not be the case in other situations. Degtiar and Rose [10] developed “conditional cross design synthesis” as an extension of Kaizar's approach, for situations when the subpopulation not included in the randomized studies is not clearly defined via an inclusion criterion. A conditional bias term is estimated based on a subpopulation that is included in both the study populations of the randomized and the observational studies. This bias term is then used to debias the estimates of the observational data.

Cross‐design synthesis relies on there being available an estimate of the target population from an observational study. This is not required by a method proposed by Dahabreh et al. [104] The method only requires that there is information about the baseline covariates in the target population. A model of treatment effect conditional on covariates is fitted based on the summary statistics from research studies of the study population, whereafter the model is used to transport an average treatment effect to the target population using information regarding their covariate distribution. Dahabreh et al. apply the method to multiple studies on hepatitis C treatments. Similar methods are presented by Zuo et al. [105] and Barker et al. [106].

Several methods exist to transport results to target populations in situations when individual participant data exist in at least one study. When using traditional meta‐analytical methods, for example, the fixed effect or random effects model, there is little gain in having access to individual participant data, as the parameter estimate will be based on means and variances of the individual samples only. Things are different when attempting to transport results to target populations. Individual participant data opens up new opportunities to take differences between sample populations into account, in particular with regard to variables that are assumed to modify the effect of the other variables on the parameter of interest. It can also be a necessary step to take when attempting to combine data from sources where the individual observations cannot be merged for legal reasons. Vo et al. [107, 124] suggest that covariate information regarding participants from several populations is used to standardize the results relative to a target population, which is used to perform a random effects meta‐analysis. Phillippo et al. [108] present a method that creates weights based on individual participant data that is used to adjust the results of studies reporting summary measures only. Yang et al. [109] propose a method to estimate treatment effect heterogeneity with a vector of known effect modifiers, based on both randomized studies and non‐randomized observation studies. Hu et al. [74] present a method to estimate the differential average treatment effect, where differences in sample populations are taken into account through propensity score modeling. Li et al. [111, 125] are interested in estimating the distribution of a variable of interest for a target population, based on samples from several populations, using selection bias modeling to account for differences between the sample populations. Han et al. [110] suggest a framework to estimate parameters for a target population based on summary statistics from heterogeneous studies. Density ratio weighting is used to account for differences in covariate levels between studies. Penalized regression is used to weight results based on sample populations, where the weights represent similarity with the target population in the distribution of relevant covariates.

### 2.3 Bayesian Models With Prior Distributions

It is common that random effect models, hierarchical models, network meta‐analysis, and meta‐regression are performed within a Bayesian framework. The Bayesian perspective assumes that the parameter of interest θ is a random variable, and the outcome of the analysis is a distribution of θ based on the available data: p(θ|X)∝L(X|θ)p(θ), where L(X|θ) is the likelihood and p(θ) is the prior distribution.

The popularity of these methods in meta‐analysis and research synthesis can be attributed to several facts. One is that the models employed in meta‐analysis and research synthesis often include parameters that have to be estimated with few data points. For instance, hierarchical models include a between‐study‐type variance parameter that has to be estimated on the available study types. When there are only a few study types, perhaps as few as two, it is difficult to estimate the variance parameter using frequentist methods. By assuming a prior distribution and employing a Bayesian perspective, a meaningful estimate can be obtained. A second reason for the popularity of Bayesian methods is that they can be used to integrate a wide range of evidence into the analysis, including expert opinion and common sense. This is usually done by formulating informative prior distributions, based on data sources that differ from the main data in some important way. We will focus on this aspect of Bayesian analysis in this section. We refer to Roos et al. [126] or Röver et al. [127] for an in‐depth overview of the impact of the choice of non‐informative or weakly informative priors.

A common situation is that the analyst is estimating the parameter θ based on a number of studies, while there are additional, less relevant studies that are also estimating θ. The additional studies can then be used to estimate the distribution of θ, and this distribution is then used as the prior distribution for the studies of main interest. Assuming that X is the data of main interest and $X^{*}$ is the less relevant data, the posterior distribution of θ given X is

$$\begin{array}{l} p(\theta |X)\propto L(X|\theta )p(\theta |X^{*}), \end{array}$$

where $p(\theta |X^{*})\propto L(X^{*}|\theta )p(\theta )$ for an uninformative prior p(θ).

In the simplest scenario, the additional studies are considered less relevant but are not assumed to differ systematically from the studies of main interest. This could be because they were performed with less rigor or on a different yet exchangeable population than the studies of main interest. Morfaw et al. [128] use both observational and randomized data on the effect of Misoprostol to prevent postpartum hemorrhage. First, the observational studies are used in a random effects model to estimate the distribution of the effect parameter. Second, the distribution based on observational data is used as an informative prior distribution in a new random effects model based on the randomized studies. A similar analysis was performed by Salpeter et al. [75] Knight et al. [76] propose to use estimates from randomized studies to create a prior distribution which is then used with longitudinal electronic medical records on the effectiveness of acetylcholinesterase inhibitors in the management of dementia, to model the trajectories of individual patients. Turner et al. [77] use estimates of between‐study variance from previous meta‐analyses to estimate the prior distribution of between‐study variance parameters. The method is applied to data comparing pharmacological interventions. Ren et al. [78] suggest that expert opinion is used to formulate a prior distribution of the between‐study variance parameter in a random effects model when the available studies are too few to estimate the parameter based on the studies alone. See also Röver et al. [129] and Lilienthal et al. [130].

Another way of using prior distributions is to adjust for biases. This can be done by adding bias parameters to the model, similar to what we saw in the section on bias adjustment. In addition, the Bayesian perspective allows the researcher to add different sorts of restrictions on the parameter of interest through the prior distribution. In doing so, a wider range of auxiliary data can be used compared to more traditional bias adjustment methods. Prevost et al. [32] and Efthimiou et al. [20] discuss how the priors can be informed to adjust hierarchical models. The prior can be designed to account for the assumption that non‐randomized studies have larger variance than randomized studies. Constraints can also be put on bias parameters, for example, by imposing that randomized studies have a smaller bias than non‐randomized studies. Morfaw et al. [128] use observational studies to create a prior distribution for the effect of Misoprostol on postpartum hemorrhage. Based on prior research, it is assumed that the estimated effect should be larger in observational studies, so the prior is constructed to give a low probability that the true effect is larger than what was shown in the observational studies. Jones et al. [89] combine studies on the effects of chemicals in drinking water, where studies report on different chemicals in humans and various species of animals. The effect of the chemicals is assumed to be different for different animals. Jones et al. discuss how to use expert opinion to model the prior distribution of the parameters describing the differences between species. A related approach is presented by Molitor et al. [131], Bartell et al. [90] use studies on the association between silica exposure and lung cancer in rats to formulate a prior distribution for the association between silica and lung cancer in humans. To allow for differences between humans and rats, a bias parameter is introduced in the prior distribution based on the rat data.

An important consideration in constructing the prior distribution based on additional studies is what weight the prior should be given in the final estimate. This weight is encapsulated in the effective sample size of the prior. With some exceptions, it is not straightforward to calculate the effective sample size. Morita et al. [123] and Neuenschwander et al. [132] offer more in‐depth discussions on this topic. In the meta‐analytical literature, a number of methods for assigning the prior a suitable weight have been suggested. One method is to introduce a new parameter in the likelihood to control the amount of information from the prior that is used. Gamalo‐Siebers et al. [39] discuss a case when the outcome is binary and can be assumed to follow the binomial distribution, with the parameter of interest θ being the probability of the outcome. The prior distribution based on additional studies is a Beta(a,b) where a is the number of events in the additional data and b is the number of non‐events. The posterior distribution of θ will then be Beta(a+x,b+n−x), with x being the number of events in the data of main interest and n being the number of participants in the data of main interest. The larger a+b is relative to n, the more impact the prior data will have on the result. A simple way of down‐weighting the prior data is to use the prior Beta(ra,rb) where r∈(0,1). r can either be set by the author or it can be estimated from the data.

When the outcome of interest is not binary but continuous, several ways of down‐weighting prior data exist: inflation factors, mixture distributions, and power priors. The methods have very similar statistical properties and differ mainly in terms of interpretation. The inflation factor method adds a parameter that controls the variance of the prior distribution. For instance, if the prior distribution follows a normal distribution, it can be formulated as

$$\begin{array}{l} \pi (\theta )∼N(\theta ,\sigma^{2}/w), \end{array}$$

where the inflation factor w∈(0,1). Schmitz et al. [40], Efthimiou et al. [20], Proctor et al. [79] apply this method to data where the prior is based on studies with populations of less interest. The mixture prior method uses a weighted combination of the prior based on the additional data and a vague prior with long tails. Assuming that $X^{*}$ is data from additional studies, a mixture prior distribution is

$$\begin{array}{l} \omega L(\theta |X^{*})(1-\omega )\pi (\theta ), \end{array}$$

where ω∈(0,1) and π(θ) is a vague distribution [72, 73].

The power prior method, proposed by Ibrahim and Chen [82], Ibrahim et al. [83], is perhaps the most influential way of downweighing additional data. Here, the additional studies are not used to inform a prior distribution. Instead, a posterior distribution is constructed based on the likelihoods of both the additional studies and the primary studies, but the likelihood of the jth prior study is raised to the power of a∈(0,1). Assuming X are the study results of main interest, $X^{*}$ are the results to be down‐weighed, a power prior estimate of the posterior distribution of θ is:

$$\begin{array}{l} L(\theta |X,X^{*},a)\propto L(\theta |X)L{\left(\theta |X^{*}\right)}^{a}p(\theta ). \end{array}$$

It is possible to use both separate power priors for each study and a uniform power prior for all additional studies. Uncertainty about a can be modeled by including a prior distribution for it. Rietbergen [84] uses the power prior for observational studies in a meta‐analysis where the prior is based on randomized studies. Hatswell [85] uses the power prior approach to downweigh studies based on publication year, sample population, and study design. This approach is applied to four distinct meta‐analyses with different topics, among them lung cancer och liver cirrhosis. Jenkins et al. [86] perform a network meta‐analysis with both RCT and observational data on relapsing remitting multiple sclerosis. Power priors are used to downweigh the observational data. Hong et al. [87] use the power prior to give more weight to studies reporting individual participant data rather than summary statistics, in an application on studies comparing a wide range of anti‐diabetic drugs. Hobbs et al. [88] propose the so‐called commensurate power prior for situations when the studies of main interest and the additional studies are assumed to estimate different parameters, that are related through $\theta_{n}|\theta_{o},\eta ∼N(\theta_{o},1/\eta )$.

### 2.4 Decision‐Centered Meta‐Analysis

The methods mentioned above extend the traditional meta‐analytical methods to better handle heterogeneity among studies. Manski [112] presents a different approach, aiming to take all relevant differences between studies into account and provide an estimated interval containing the parameter of interest. Manski considers

$$\begin{array}{l} E(y|x_{0}), \end{array}$$

where y refers to a clinical outcome and $x_{0}$ refers to a vector of patient characteristics. While Manski uses a slightly different theoretical framework than Rubin, $E(y|x_{0})$ can be understood in terms of external and internal biases. When studies report estimates that differ from y and $x_{0}$, for example, because different populations are studied or surrogate outcomes are studied, the studies are not providing the point estimate of interest, but biased estimates. To take the biases into account, Manski proposes that we should not use the point estimates from the j‐th study, but rather an interval of values $H_{j}$ that can be assumed to include $E(y_{0}|x_{0})$. This interval is obtained by using information about internal and external biases, and making plausible assumptions based on discussions with subject‐matter experts. Similar intervals are obtained based on all m studies included in the meta‐analysis. An interval of plausible values is then calculated as the intersection of all m intervals:

$$\begin{array}{l} H_{0}=\cap_{j\in \{1,\ldots ,m\}}H_{j}. \end{array}$$

If $H_{0}$ is the empty set, the studies were flawed. Presumably, $H_{0}$ could also be the empty set because the assumptions used in creating the intervals were unrealistic. However, Manski does not explicitly say so.

Manski illustrates the method with an application involving four studies investigating the effectiveness of a treatment against high blood pressure. While the target population is US residents who could potentially be treated against high blood pressure, three of the studies are randomized trials based on samples from non‐American populations. The fourth study is an observational study based on USA residents. Manski argues that the randomized studies suffer from external biases (e.g., because the study populations consist of non‐US residents) but have few internal biases. The observational study suffers from internal biases (e.g., little is known about the treatment assignment processes), but it has few external biases. For each study, an upper and lower limit is constructed based on the reported mean effect and an assumed maximum contribution of the biases. This results in wide intervals for each study, which can be assumed to contain $E(y|x_{0})$. The intersection of the intervals yields an interval $H_{0}$ which is much smaller.

## 3 Relationships Between the Methods

**FIGURE 1:**
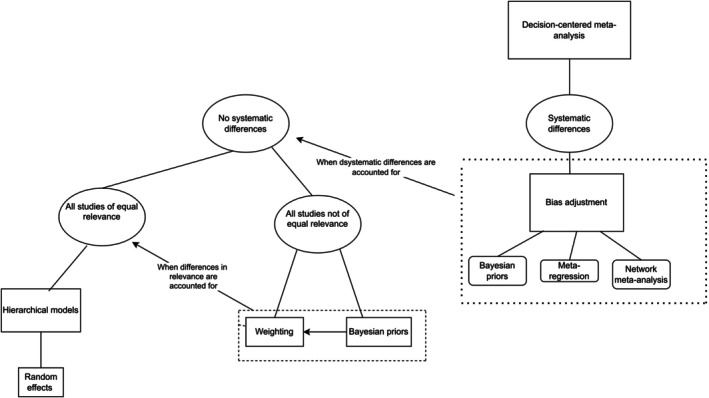
Illustration of the relationship between the methods.

The methods described in this article lend themselves to solving different problems, and they can be combined to summarize studies that differ in various ways. This section describes the relationship between the methods. We first describe what methods are applicable when the studies included in an analysis differ in systematic ways, and then what methods are applicable when the differences between studies are not systematic. Finally, we describe how methods can be combined when both systematic and non‐systematic differences exist between the studies. Figure 1 illustrates the relationships.

Figure 1 can be interpreted as a decision‐tree. A set of studies to be summarized may differ in several regards that can be accounted for by applying the methods discussed in the review article.

If a set of studies have systematic differences, these can be taken into account through various bias adjustment methods described in the review, including Bayesian riors. When that is done, there are no systematic differences, but the studies may still be of unequal relevance. This may be accounted for by weighting or Bayesian priors.

When the studies are of equal relevance and no systematic differences remain, differences between studies that are difficult to model may still exist, e.g., because studies use different experimental designs. Hierarchical models, including random‐effects models, may then be used.

### 3.1 Systematic Differences Between Studies

Bias adjustment, network meta‐analysis, and meta‐regression are suitable when the collected studies have systematic differences, that is, when the studies are non‐exchangeable, either in the form of external or internal biases. However, the methods rely on different assumptions and are plausible in different situations.

Meta‐regression is plausible when the relationship between the systematic differences and the parameter estimate is assumed to be linear, and network meta‐analysis is suitable when the systematic differences are due to a network of effect comparisons. In both cases, an estimate of the effect that the systematic differences are having is given by the model, based on the studies of main interest. However, both methods rely on there being available a relatively large number of studies with similar properties, which is often unrealistic. The bias adjustment methods discussed in this review are helpful in trickier situations, when the differences between studies are harder to model, for instance, because of a lack of sufficient studies. Instead of attempting to estimate the effect of systematic differences on the parameter of interest, they account for the systematic differences by using a broader range of data, for example, expert opinion and results from completely different study designs or even different fields of study. The risk is, obviously, that the adjustments are not valid for the studies of main interest. This adds a layer of uncertainty to the results, and as we have seen several examples of, this uncertainty is often handled by using a Bayesian framework, where the assumptions of the bias term can be modeled via a prior distribution. The Bayesian methods offer additional flexibility, in that biases can be modeled in many different ways, and that a wide range of information can be used to inform the prior distributions, for instance when external evidence is used to generate a prior distribution that puts a limit on the difference in treatment effect between two study types [31, 98]. As is always the case with Bayesian methods, the subjective opinions of the analyst play a key role, and a risk is that the results reflect the analyst's opinions and preconceptions.

### 3.2 No Systematic Differences Between Studies

When there are no systematic differences between studies, meaning that they are exchangeable, methods can be used to account for the differences in level of uncertainty that different studies have. We have seen that methods distinguish between two situations: when all studies are equally relevant and when studies differ in terms of relevance.

Studies differ in terms of relevance, for instance, when some studies suffer from internal biases that yield the less reliable results. Weighting and Bayesian priors are two ways of taking relevance into account. Weighting does this by down‐weighting less relevant studies. Bayesian methods can use the less relevant studies to calculate a prior distribution, which in most cases simply means that less relevant studies are given less weight. With either method, the relative weight to be assigned to different studies relies to a large degree on the subjective judgment of the analyst, who has to decide what counts as a “less relevant” study based on a wide range of considerations and quantify what impact such a study should have. As we have seen, it is usually difficult to find reliable data to base the weights on. This creates additional uncertainty in the results. A benefit of the Bayesian perspective is that this uncertainty is part of the model through the specification of the prior. On the other hand, practitioners who avoid the Bayesian framework may have a stronger reason to search for valid external evidence to base weights on. It can be added that the standard random effects and fixed effect models of meta‐analysis are also down‐weighting with larger sampling variance. What makes the weighting methods and Bayesian priors discussed in this article different is that the weights are based on other considerations than sampling variance only.

If studies do not differ in terms of relevance and no systematic differences are assumed, that is, exchangeability is assumed, the studies are usually assumed to differ in various ways that cannot realistically be modeled. Hierarchical random effects models are used when the studies are clustered, for example, based on study design or sample populations. If no clusters are assumed, a standard random effects model can be used. If it is believed that all differences between studies are properly taken into consideration, a fixed effect model can be used. This, however, is rarely the case.

### 3.3 Combining Methods

Often, studies differ from each other in both systematic and non‐systematic ways. The methods discussed in this article can then be combined to take several differences into consideration. One example is when some studies suffer from biases such that they are expected to over‐ or underestimate the parameter of interest. Bias adjustment can be used to correct for this. The bias remains a problem to the extent that it introduces more uncertainty regarding the estimates, making some studies less relevant than others. Weighting methods can be used to account for this [7]. Alternatively, once study results are adjusted for biases, they may be of similar relevance, and a hierarchical model or random effects model can be used with the bias‐adjusted estimates [4, 15, 32].

In other situations, the systematic differences can be accounted for with meta‐regression or network meta‐analysis. After this, non‐systematic differences may remain. These differences can be accounted for with Bayesian methods, weighting schemes, or hierarchical models. For instance, meta‐regression and network meta‐analysis may be defined in a random effects or a hierarchical framework [34, 133, 134]. There are also examples of meta‐regression and network meta‐analyses that employ weights or prior distributions based on external data [68]. When studies do not differ in systematic ways but do differ in terms of relevance, Bayesian priors or weights can be used to account for the difference in relevance, within a hierarchical model or a random effects model [63].

It is rare to see articles where the authors explicitly consider differences between studies along more than one or two dimensions. In their description of cross‐design synthesis, the GAO [98] explicitly mentions that when transporting results to a population, other biases between studies have to be adjusted for. No applied example is given, however. In this regard, the confidence profile method, suggested by Eddy et al. [121], can serve as inspiration. It was presented at the infancy of research synthesis methods, and it combines several of the methods discussed in this article. It is formulated within a Bayesian framework, and the foundation of the method is to adjust the likelihoods of all included studies for the biases that are assumed to exist. In addition, the authors add more methods. Eddy et al. give examples of network meta‐analysis to combine results of different interventions and chains of evidence analysis to consider different outcomes; the book contains an example of hierarchical models to account for differences in study population, and it contains examples of auxiliary information used in formulating prior distributions.

## 4 Discussion

The purpose of this review was to identify and describe methods to account for differences between studies included in a meta‐analysis. Outside of the already well‐established methods, random effects models, network meta‐analysis, and meta‐regression, we have identified six categories of methods and discussed the sorts of bias they are suitable for, and how they can be combined to account for differences between studies along multiple dimensions. Nevertheless, the vast majority of meta‐analyses published use traditional methods that may overlook differences between studies. One reason for this may be that most meta‐analyses are written not with the aim of obtaining an estimate for a target study, but rather to give a summary measure of existing research.

Another reason is that the methods covered in this article are probably more difficult to apply than the traditional methods, which are available in most statistical software packages. This point echoes some of the earlier reviews of this field [9, 15]. There may not be an easy remedy to this problem. Taking differences between studies into account and determining what auxiliary evidence is useful is not computationally demanding, but it does require that the analyst considers the differences between studies, which is time‐consuming and requires a deep subject‐matter knowledge.

A topic that this review has not covered to a large extent is the situation when there are individual participant data available from at least one study. As we saw in the section on sample selection bias, this opens up opportunities to draw more elaborate conclusions that take into account differences between different populations. Surely, individual participant data can be used to make adjustments for biases other than sample selection bias.

## Funding

The authors have nothing to report.

## Ethics Statement

The authors have nothing to report.

## Consent

The authors have nothing to report.

## Conflicts of Interest

The authors declare no conflicts of interest.

## Data Availability

The authors have nothing to report.
